# Deep neural networks based automated extraction of dugong feeding trails from UAV images in the intertidal seagrass beds

**DOI:** 10.1371/journal.pone.0255586

**Published:** 2021-08-13

**Authors:** Chiaki Yamato, Kotaro Ichikawa, Nobuaki Arai, Kotaro Tanaka, Takahiro Nishiyama, Kongkiat Kittiwattanawong

**Affiliations:** 1 Graduate School of Informatics, Kyoto University, Kyoto, Japan; 2 Field Science Education and Research Center, Kyoto University, Kyoto, Japan; 3 Graduate school of Agriculture, Kyoto University, Kyoto, Japan; 4 Phuket Marine Biological Centre, Phuket, Thailand; San Diego Zoo Institute for Conservation Research, UNITED STATES

## Abstract

Dugongs (*Dugong dugon*) are seagrass specialists distributed in shallow coastal waters in tropical and subtropical seas. The area and distribution of the dugongs’ feeding trails, which are unvegetated winding tracks left after feeding, have been used as an indicator of their feeding ground utilization. However, current ground-based measurements of these trails require a large amount of time and effort. Here, we developed effective methods to observe the dugongs’ feeding trails using unmanned aerial vehicle (UAV) images (1) by extracting the dugong feeding trails using deep neural networks. Furthermore, we demonstrated two applications as follows; (2) extraction of the daily new feeding trails with deep neural networks and (3) estimation the direction of the feeding trails. We obtained aerial photographs from the intertidal seagrass bed at Talibong Island, Trang Province, Thailand. The F1 scores, which are a measure of binary classification model’s accuracy taking false positives and false negatives into account, for the method (1) were 89.5% and 87.7% for the images with ground sampling resolutions of 1 cm/pixel and 0.5 cm/pixel, respectively, while the F1 score for the method (2) was 61.9%. The F1 score for the method (1) was high enough to perform scientific studies on the dugong. However, the method (2) should be improved, and there remains a need for manual correction. The mean area of the extracted daily new feeding trails from September 12–27, 2019, was 187.8 m^2^ per day (n = 9). Total 63.9% of the feeding trails was estimated to have direction within a range of 112.5° and 157.5°. These proposed new methods will reduce the time and efforts required for future feeding trail observations and contribute to future assessments of the dugongs’ seagrass habitat use.

## 1 Introduction

Dugongs (*Dugong dugon*) are herbivorous marine mammals found in the shallow coastal waters of tropical and subtropical seas. They are seagrass specialists, feeding almost exclusively on phanerogams from the families of Potamogetonaceae and Hydrocharitaceae [1–4]. Their numbers are decreasing mainly due to accidental bycatches in coastal gillnet fisheries and declining seagrass habitats [5]. Consequently, they have been listed as vulnerable on the IUCN Red List since 1982 [6]. In multiple habitats worldwide, the seagrass beds that the dugongs feed on have been made into sanctuaries, which prohibits the operation of fisheries and vessel passage [5]. Sustainable conservation, however, should aim to minimize the suppression of local people’s lives, such as those who operate coastal fisheries. Improved knowledge about dugong feeding behaviors would help limit these sanctuaries spatially and temporally, thus minimizing the suppression of the local fisheries.

Numerous factors can influence dugong feeding ground selection, including external ones such as the possibility of stranding and human disturbance, physical characteristics such as sediment type, depth, and tidal currents, or biological factors such as seagrass species, the biomass above and below ground, digestibility, and the nutrient contents (including nitrogen and soluble carbohydrates) [1, 3, 4, 7–12]. Theoretically, dugong feeding selection is assumed to be based on the maximum energy gained with minimal energy expended [4, 10, 11]. These reports are based on the analysis of stomach contents, fecal samples, mouth samples, global positioning system (GPS) telemetry, and observations of their feeding trails, which are unvegetated winding tracks left after they have fed.

Previous studies using GPS telemetry indicated that tidal currents are one of the factors that may influence dugong feeding migration [13]. The use of tidal current transport is well documented in marine animals and such transport is considered energetically beneficial [14]. It is possible that tidal currents are the most important determinant of their feeding ground selection, as is the case for the green turtle [15]. However, because GPS telemetry only provides time and location data, there is a lack of information regarding the dugongs’ detailed activity (i.e., if the dugongs feed, and if so, to what extent).

Observing the feeding trails of dugongs is an effective method to monitor their feeding activity in detail. Dugongs uproot entire plants (including rhizomes and roots) [3, 16] and the leaves, rhizomes, and roots of the seagrasses are exposed at the edges of their feeding trails. Their width, length, and depth are generally between 10 and 25 cm; 30 cm and several meters [8, 13]; and 2.6 and 3.6 cm [17], respectively. They provide direct evidence of feeding and information about the location of feeding and seagrass consumption, and feeding direction. Intertidal seagrass beds specifically, which are exposed at low tides, are considered important feeding grounds [18–21]. In Thailand, both visual and acoustic observations suggest that dugongs feed significantly and more frequently in the intertidal seagrass beds than in the subtidal seagrass beds [19, 20]. In addition, intertidal seagrass beds are the optimal fields for the observation of feeding trails because they are exposed, allowing a wider observation range when compared to that by scuba diving.

Previous observations on feeding trails have been conducted mainly on the intertidal seagrass beds and they have clarified that dugongs prefer seagrass beds that are higher in nitrogen and soluble carbohydrates (starch) and lower in fiber [4, 12, 17, 19, 22–27]. Seagrass recovery rate [24, 25] and seagrass consumption [26] have also been studied in intertidal seagrass beds. Information about seagrass recovery and consumption is important to estimate the carrying capacity of the seagrass beds. Seagrass consumption by the dugongs is estimated by measuring the areas of the feeding trails made in a certain period and the seagrass biomass removed from that area [26, 28]. However, ground-based observations of the feeding trails in the intertidal seagrass beds require large amounts of time and labor so that the observation areas tend to be small-scale and dispersive.

Unmanned aerial vehicles (UAV) have shown rapid usage as a tool for reducing the time and labor of the ground-based observations. The cost of UAV monitoring is low in comparison with other observation methods which use airplanes or helicopters. Therefore UAV is used in various application in aquatic wildlife science; monitoring the presence of animal [29], monitoring fine-scale behavior [30, 31], population assessment [32–34], individual identification [35–37], measurement of body length and mass [35, 38, 39] and habitat mapping [40]. Some of these applications use a photogrammetric approach that provides a flat and undistorted field of view [35, 38–40]. This approach is increasingly being utilized in the context of seagrass mapping [41–44]. However, few applications in high-turbidity waters have been reported [44] because the performance of photogrammetry in such areas is affected by optical properties of water including turbidity and sunlight reflection [45–47].

UAV photogrammetry allows to expand observation area of dugong feeding trails, however, the process of extracting feeding trails from the aerial images is labor-intensive. Automation of the extraction process will improve the efficiency of analysis, but the following challenges must first be overcome:

1. Feeding trails vary in their (i) shape, (ii) color, and (iii) illuminance. (i) They wind irregularly and do not have a constant width. (ii) The exposed seagrasses at the edges of the feeding trails are covered with sediment transported by the daily tidal currents. Therefore, the color of the feeding trails becomes similar to that of the sediments as time passes. Furthermore, the visible duration of the feeding trails in the seagrass beds dominated by *Halophila ovalis* is reported to be 8.5 ± 0.33 days [28]. (iii) The ground surfaces of the feeding trails are excavated to depths of around 3 cm [17] and pools of water are left at low tide. Sunlight reflection on the pools of water produce highlight on the images and affects illuminance of feeding trails. Since the intensity of sunlight reflection is influenced by cloud cover and solar altitude, the illuminance of feeding trails varies according to the photographing time.
2. The amount of the ground-truth datasets was limited. This was because the observation area was limited. The ground-based observations are labor-intensive and are required to be conducted during low tides when the seagrass bed was exposed in air.

To deal with challenge (1), deep neural networks are employed. Deep neural networks use automated parameter optimization to extract the features of objects using colors, textures, and shapes of possibly segmented, meaningful regions in the image, thus enabling classification with high versatility [48]. Deep neural networks are also used in some cases of seagrass mapping [49, 50]. Perez [49] applied deep neural networks to quantify seagrass distribution based on multiband satellite images (ground sample distance of 1.24 m) and showed that deep neural networks achieved much better results than a linear regression model and a support vector machine did. Weidmann [50] applied deep neural networks to seagrass segmentation based on images taken by an autonomous underwater vehicle (AUV). They achieved a mean intersection over union of 87.87%. Among the multiple deep neural networks, encoder-decoder architectures represented by U-Net [51], which are a so-called fully convolutional network, are supposed to mitigate challenge (2). It is computationally efficient and delivers good results with small amounts of ground-truth data [51–53], and therefore, is being increasingly employed to analyze high-resolution data collected using UAV [54–58].

The purpose of this investigation was to establish an efficient method to observe dugong feeding trails in the intertidal seagrass beds. In this study, we propose a workflow for data acquisition from UAV, using the automated extraction of feeding trails based on deep neural networks and an estimation of feeding directions using the results of extraction.

## 2 Materials and methods

### 2.1 Overview of the proposed method

**Fig 1:**
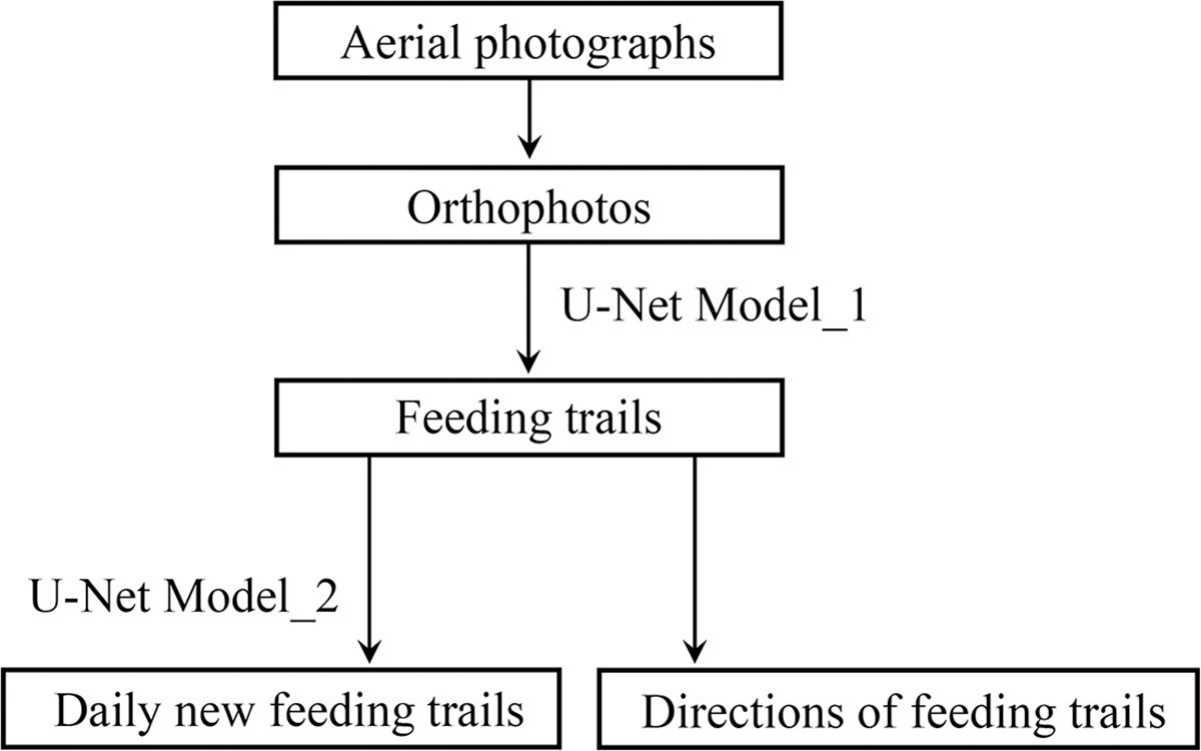
Flowchart of the proposed method. In this paper, “daily new feeding trails” were defined as the feeding trails that were made after the image acquisition with UAV on a certain day and before the next image acquisition on the following day. Furthermore, “Model_1” and “Model_2” were developed by transfer learning from U-Net to extract the feeding trails and the daily new feeding trails, respectively.

The flowchart of the proposed method is shown in Fig 1. The aerial photographs of an intertidal seagrass bed were obtained using UAV once a day when weather and tidal conditions allowed. Then, orthophotos which are geometrically collected aerial images were generated from the aerial photographs. The feeding trails were extracted from the orthophotos with a deep neural network developed by transfer learning based on U-Net [51]. Hereinafter, this model is called “Model_1”. Then, two applicable analyses of the extracted feeding trails were demonstrated. One was to extract “daily new feeding trails.” In this paper, “daily new feeding trails” were defined as the feeding trails that were made after the image acquisition with the UAV on a certain day and before the next image acquisition on the following day. They were extracted from the differential images of the extracted feeding trails generated from any given two consecutive days by the Model_1. Then another U-Net-based model (hereinafter referred to as “Model_2”) was trained to extract the daily new feeding trails from the differential images. The second was to estimate the directions of the feeding trails.

### 2.2 Field data collection

**Fig 2:**
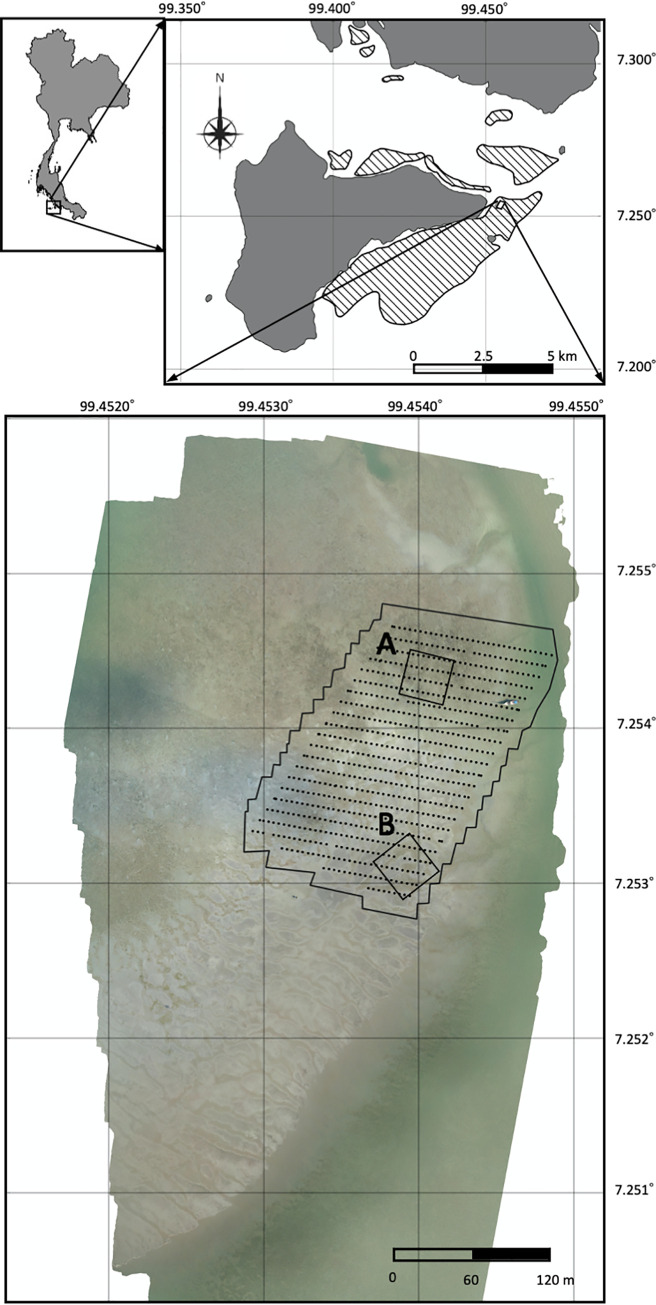
Map (upper inset) and orthophoto (bottom inset) of the study site. Striped area in the upper inset indicates the seagrass distribution (Kittiwattanawong personal communication). In the bottom inset, the black line and black points show the aerial observation area and the camera positions, respectively. A) and B) show the ground-truth observation areas of 30 m × 30 m.

Field surveys were conducted from September 2 to October 1, 2019, in the intertidal seagrass bed on the east of Talibong Island, Trang Province, Thailand (Fig 2). This study was approved by the Animal Experimentation Committee of the Graduate School of Informatics, Kyoto University (Approval number: Inf-K19003). All aerial observations were conducted under the regulations of the Announcement of the Ministry of Transport on Rules to Apply for Permission and Conditions to Control and Launch Unmanned Aircraft in the Category of Remotely Piloted Aircraft B.E. 2558, Published in 2015, and with the permission of the National Broadcasting and Telecommunications Commission (Registration number: 030962-16-0001).

The most dominant seagrass species of the seagrass bed was *Halophila ovalis*, followed by *Cymodocea rotundata*, *Cymodocea serrulata* and *Enhalus acoroides*. Approximately 120–150 dugongs were estimated to inhabit this area, representing the largest population in Thailand [59, 60]. Most of the population appeared to use this seagrass bed as a feeding ground ([19, 59]; Kittiwattanawong, personal communication). All the observations were conducted over a period of 1.5–3 hours during low tides when the seagrass bed was exposed in air.

We selected the aerial observation area (about 250 m × 150 m; 3.6 ha) and two ground-truth observation areas (30 m × 30 m square quadrats), that included many new feeding trails with which to test the algorithm. Both ground-truth observation areas were set inside the aerial observation area; plot A (07°15′15.19″N, 99°27′14.37″E) and plot B (07°15′11.19″N, 99°27′13.91″E). Fluorescent pink ribbons were set at 5-m intervals as markers on the aerial images. The length, width, and position of each feeding trail were measured to ± 0.5 cm accuracy using rulers and/or tape measures. The ground-truth observations were conducted every day, except for rainy days and on neap tide days. On neap tide days, the intertidal seagrass bed did not dry out at all during the day. The ground-truth observations were not conducted on neap tide days as it was hard to recognize submerged feeding trails because the turbidity level was high in this area. New feeding trails that were found in the ground-truth observation areas were also recorded during the ground-truth observation period.

In tandem with the ground-truth observations, aerial observations were conducted by taking photographs with a Phantom 4 PRO V2 (DJI, Shenzhen, China). A single-grid flight course was programmed with GS PRO (DJI, Shenzhen, China), and the flights were operated automatically. We deployed plastic boards (0.5 m × 0.5 m) as ground control points (GCPs) to improve the quality of the orthophotos; they were used to georeference the orthophotos. The flights were carried out once a day from September 12 to 27, 2019, except during rains. During each flight, 4 or 5 GCPs were set to be widely distributed across the aerial observation area [61, 62], and their positions were measured using a handy GPS (GPSMAP 64sc J, Garmin, Kansas, USA). The coordinates of the two ground-based observation areas were also recorded. The positioning errors of the latest model (GPSMAP 66) of this handy GPS obtained with GPS satellites in a low multipath environment are estimated to be 1.51, 1.11, and 2.64 m in latitude, longitude and altitude, respectively, for the single point mode and 0.14, 0.08, and 0.26 m, respectively, for the differential mode [63]. Approximately 750 aerial photographs were captured each day. The ground sampling resolution and the distance from the ground were 1.0 cm/pixel and approximately 37.7 m from September 12 to 13, and 0.5 cm/pixel and 18.9 m from September 15 to 27, respectively. On September 14, flights were not conducted because of rain. The camera positions are represented as black points in Fig 2. The frontlap and sidelap of the images were 80% and 70%, respectively.

### 2.3 Orthophoto generation

Orthophotos were generated using the Structure-from-Motion Multi-View Stereo (SfM-MVS) algorithms. SfM-MVS algorithms are increasingly utilized to generate high-resolution three-dimensional (3D) models in the field of habitat mapping [40, 64]. Metashape Professional Edition v1.5.4 (Agisoft LLC) was used for the processing. It is a commercial software that performs 3D reconstruction of objects based on SfM-MVS algorithms. First, coordinates of the GCPs and the ground-truth observation areas were used for georeferencing [65]. Then, camera parameters and orientations were calculated from multiple aerial photographs from different positions, and a sparse 3D point cloud representing the most prominent features in the images was generated using SfM algorithms [66]. After that, a 3D dense point cloud representing the object’s surface geometry was generated using MVS algorithms. Finally, orthophotos were constructed based on the 3D dense point clouds. In the subsequent analysis, the resolution of the data was unified into 0.47 cm/pixel.

### 2.4 Automatic extraction of the feeding trails

#### 2.4.1 Learning deep neural networks based on U-Net

**Fig 3:**
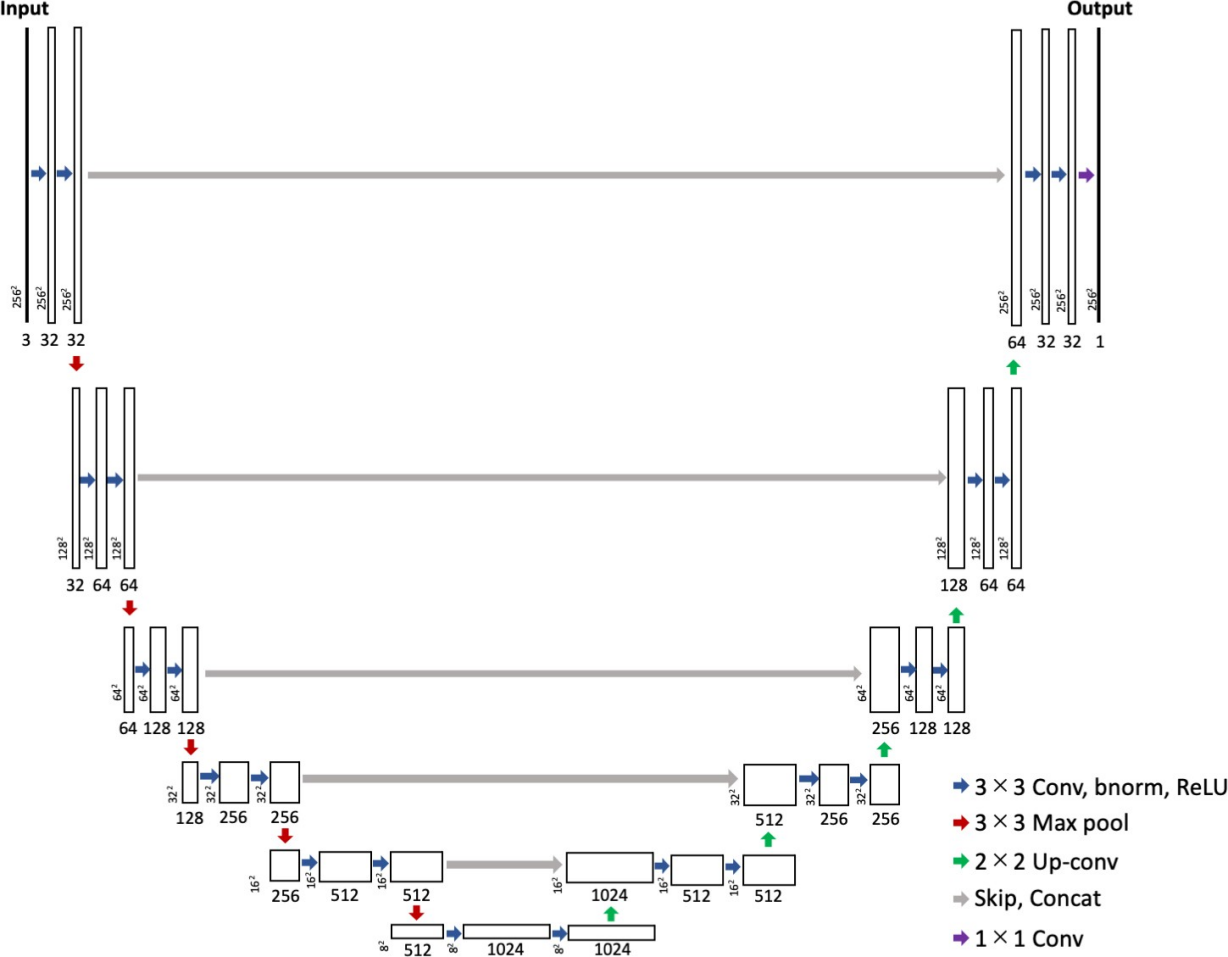
The network architecture used for the extraction of the feeding trails. The left part of the figure shows down-sampling operation and right part shows up-sampling process. Each box corresponds to a multi-channel feature map. The number of channels is denoted on the bottom of the box. The x-y-size is provided at the lower left edge of the box. The blue arrow shows convolution operations; the convolution with a 3 × 3 kernel size, followed by the batch normalization, leaky rectified linear units (ReLU) activation. The red arrow shows max-pooling layer operation with the window size of 2 × 2, and the green arrow shows 2 × 2 up-convolution. The gray arrow shows the skip connection in which the feature of the encoder layer is copied and then concatenated to the result of the deconvolution. The resulting feature layer is generated by the 1 × 1 convolution (purple arrow).

The U-Net [51] architecture was utilized to extract the feeding trails from the orthophotos. The architecture builds upon the fully conventional networks [51, 67]; the most successful state-of-the-art deep learning techniques for semantic segmentation [68]. This consists of encoder-decoder architecture and adds additional skip connections between the layers at the same hierarchical level as in the encoder and decoder. This allows for the precise localization of the features in the image-reproduction stage. Fig 3 outlines the network architecture used in this study. It includes an extra pair of encoder and decoder layers. The number of layers was selected based on a preliminary test, in which precision and recall were verified for a small dataset (consisting of images of plot A obtained on 15th September). Each encoder layer consists of a strided 2D convolution of stride 2, batch normalization and leaky rectified linear units (ReLU). In the decoder, we use a strided deconvolution of stride 2, batch normalization, and ReLU. In the final layer, we used a sigmoid activation function. Given the heavy computational requirements of training such a model, the sampling rate of the input images was set to 256 × 256 pixels.

**Fig 4:**
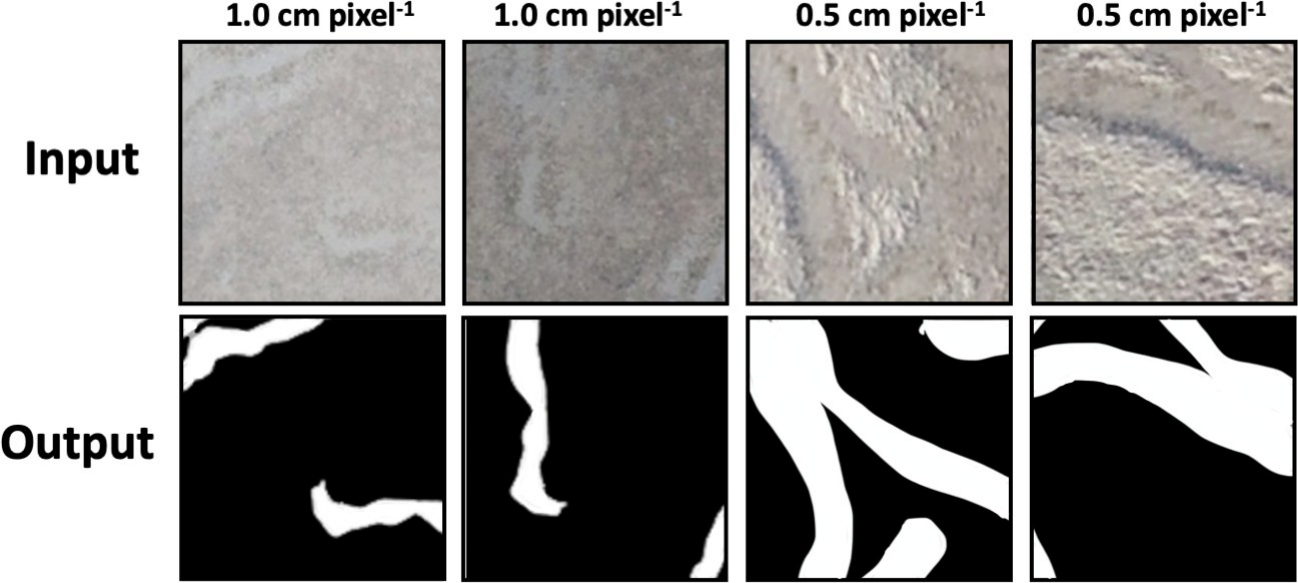
Examples of the training data. Upper insets show the input images and the lower insets show the output images.

The training data set for the Model_1 was made by manual annotation of the orthophotos referring to the records of the ground-based observations (Fig 4). It was comprised of the orthophotos from 5 days. The orthophoto of the observation area from each day (6400 × 6400 pixels) was split into 9313 blocks (256 × 256 pixels). To enhance the size of the data set, each block was cropped to be overlapped by 75% (192 pixels) with the neighboring images. Only orthophotos of plot A were adopted because the seagrass cover in plot B was so low that it was difficult to identify each trail by visual inspection, both on-site and off-line (Table 1). Only visible feeding trails on an orthophoto were annotated among feeding trails. In addition to the images of the ground-based observation area, the edges of the orthophotos were added to the data set.

**Table 1:** Training data set for Model_1.

Photographing date (/2019)	Area of orthophoto	Resolution (cm / pixel)
9/12	PlotA	1
9/13	PlotA	1
9/15	PlotA	0.5
9/15	Edge	0.5
9/17	PlotA	0.5
9/25	PlotA	0.5

The data set comprised of orthophotos of plot A and edge of the observation area.

To increase the diversity of the data available for the training of the machine learning model and to avoid overfitting, data augmentation was performed [69]. The cropped images were randomly augmented by rotation (-90, 0, 90, 180), flip, gamma variations (0.6–1.2), and contrast variations (0.8–1.4).

The training dataset was split into a training set (50%) and a validation and test set (50%). The initial learning rate was 0.01, and the parameters were optimized using the Adam algorithm [70].

#### 2.4.2 Extraction of the feeding trails

**Fig 5:**
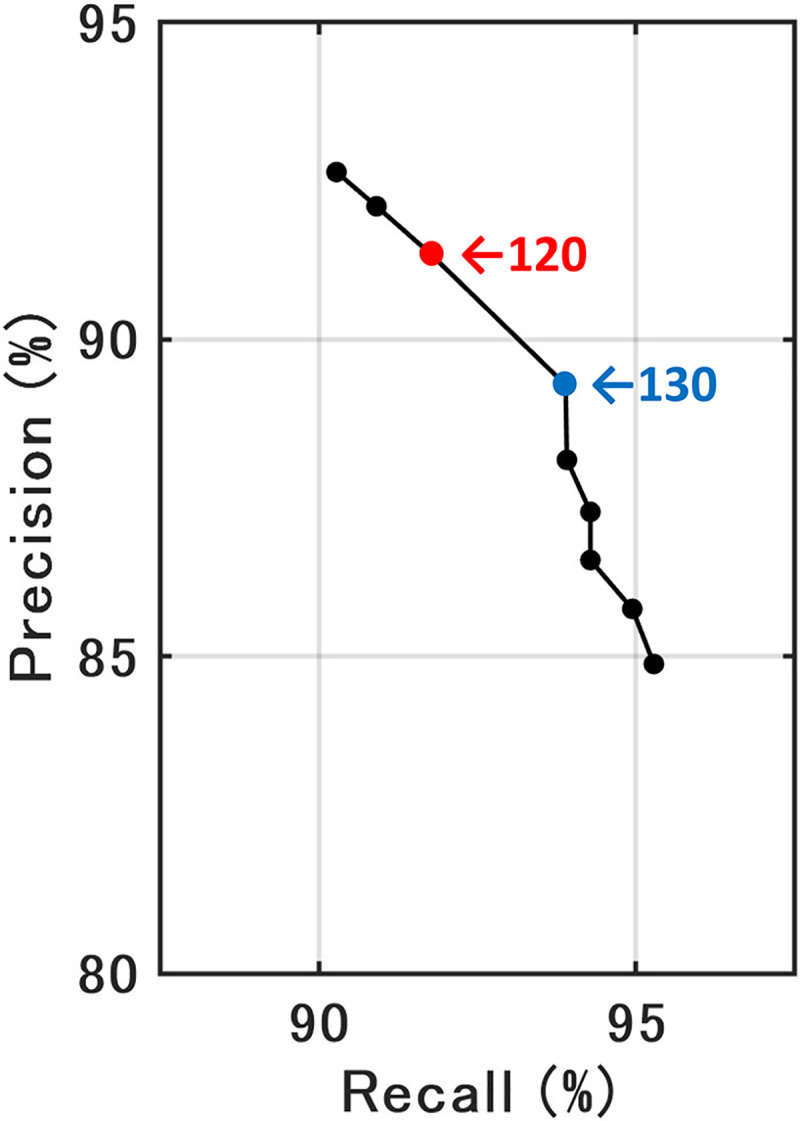
Precision-recall curve at various luminance threshold values for binarization of the integrated image. The red and blue arrows highlight the optimum values.

The orthophoto of the aerial observation area was divided into 256 × 256 pixels blocks to be input into the learned Model_1. Each block was overlapped by 50% (128 pixels) and 4 predictions were performed except on the edge of the aerial observation area. The predicted results were integrated and binarized using a luminance value threshold. To evaluate the optimum threshold value of luminance, the threshold was verified by step of 10 from 100 to 180 for a small dataset (consisting of images of plot A obtained on 15th September). A precision-recall curve was drawn, and the optimal value was determined to be 125 by visual inspection (Fig 5).

#### 2.4.3 Evaluation

The F1 score was used to assess the accuracy of the model, as it is generally used for evaluations in the field of pixel-based extractions. It was calculated based on the true positives TP (pixels extracted correctly as feeding trails), false positives FP (pixels extracted incorrectly as feeding trails), and false negatives FN (pixel extracted incorrectly as not feeding trails). Since the F1 score is defined as (Eq 1), the harmonic average of the precision is defined as (Eq 2), and the recall which is defined as (Eq 3), the F1 score was an ideal metric for the evaluation of both the precision and recall at the same time [71].


$$F1\;score=\frac{2TP}{2TP+FP+FN}$$ (1)

$$Precision=\frac{TP}{TP+FP}$$ (2)

$$Recall=\frac{TP}{TP+FN}$$ (3)

**Fig 6:**
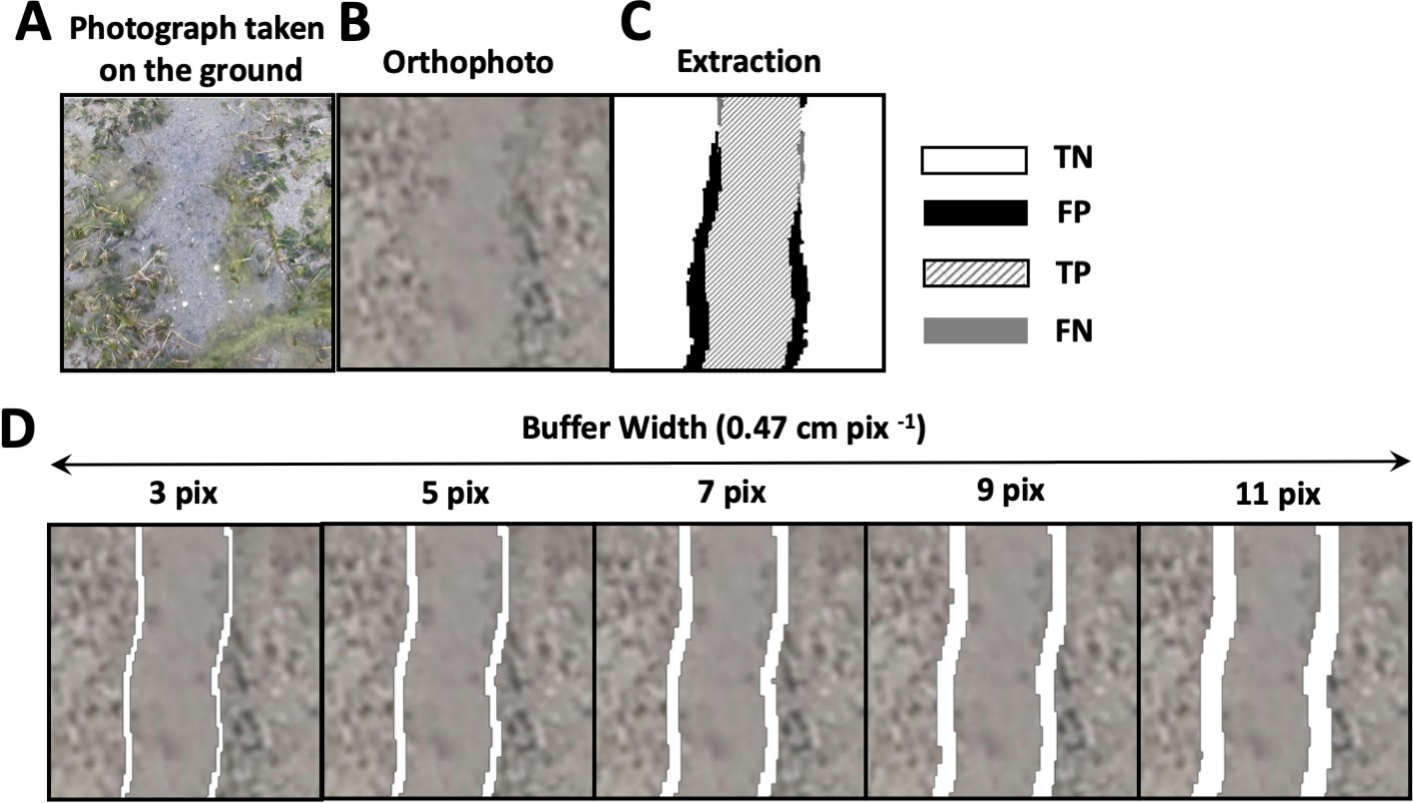
Reference diagrams for the buffer widths. (A) an example image of typical feeding trails taken during ground-based observations; (B) an example of the feeding trails on an orthophoto; (C) an example of the extracted feeding trails; and (D) the buffers for different widths. The white area in (D) shows the buffer.

Considering the possible annotation errors, we set a buffer for the boundary areas of the feeding trails. In the ground-truth observations, the observers decided the boundary of the feeding trails mainly by using the leaves or roots of the seagrass that were exposed at their edges (Fig 6A). However, the leaves or roots were difficult to locate, even in the orthophotos (Fig 6B) with a ground sampling resolution of 0.5 cm/pixel. This means that manual annotation error may occur (Fig 6C).

The buffer was defined as the difference between the annotated feeding trails and those after dilation operation. Within the buffer, all the pixels of extracted feeding trails were classified as true positives TP. For the purpose of evaluating the optimum width of the buffer *w*, the width of the square-shaped structuring element (*w*+2) of the dilation operation was verified by 1 pixel (Fig 6D). The structuring element was a small matrix used in morphological processes, including dilation. In the dilation operation, the structural element was positioned at all possible locations in the image, and its origin (the center of the shape) was set to 1 if any of its corresponding neighborhood pixels were also 1.

**Fig 7:**
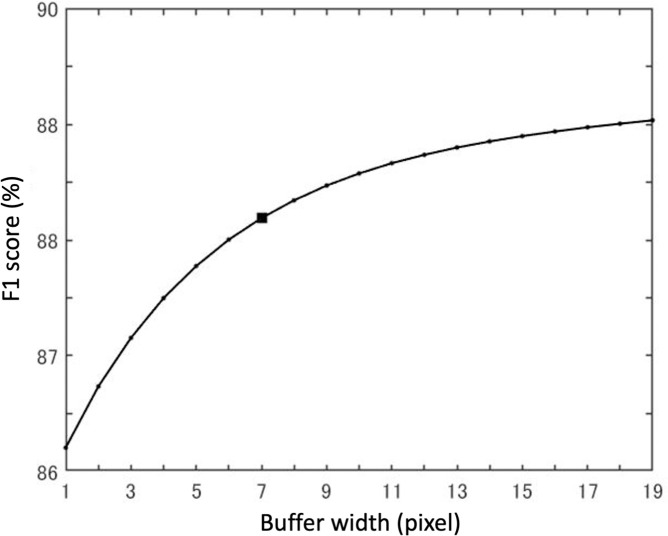
F1 score of the feeding trails extracted with different widths of the buffer. The black point indicates the changing point at an incremental rate.

The F1 scores for the Model_1 were increased by extending the buffer width (Fig 7). Incremental rates of the F1 scores with the extending buffer width were degraded when the width exceeded 7 pixels. The buffer width was set to 7 pixels considering that substantial annotation errors at the boundary areas for the feeding trials were allowed within this width. The width was equivalent to 3.25 cm, which is 25% of the average width of the feeding trails observed in this study (13.00 cm, n = 1352).

### 2.5 Automatic extraction of the daily new feeding trails

#### 2.5.1 Overview

**Fig 8:**
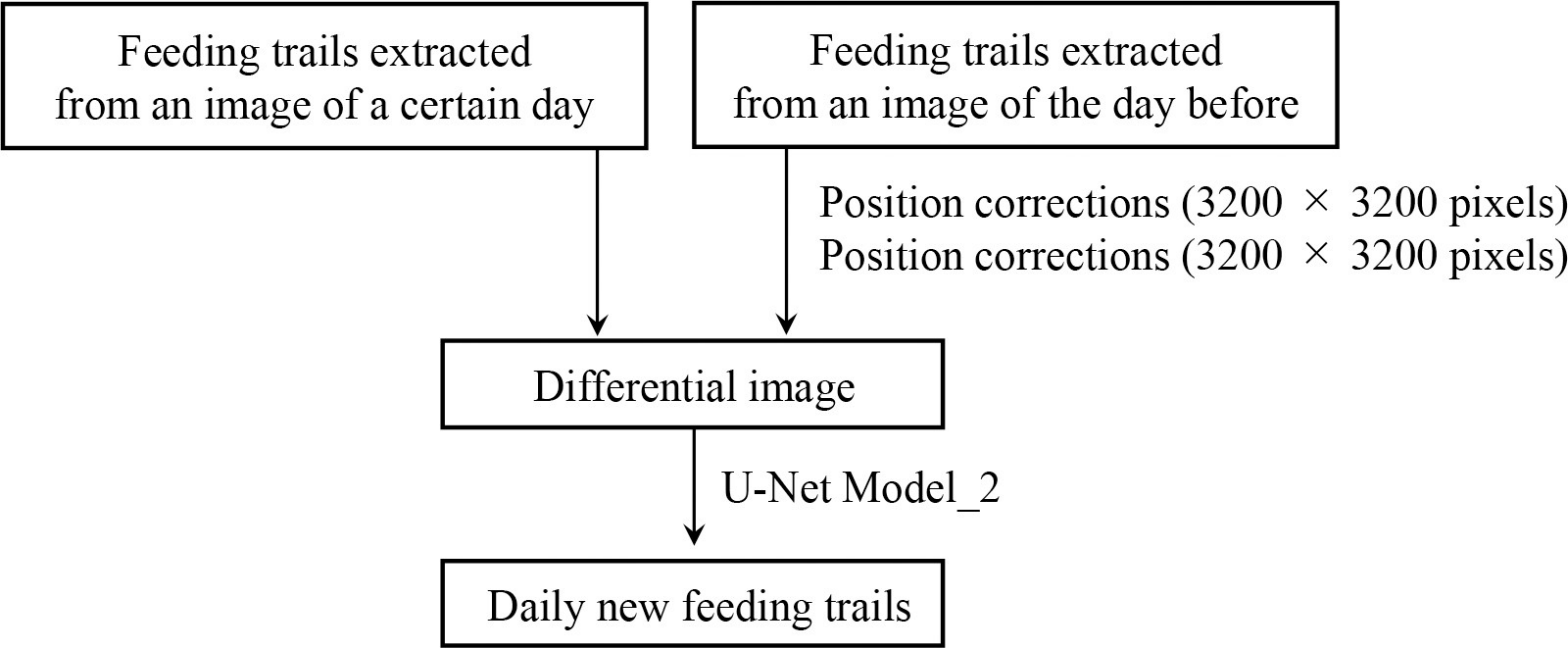
Flowchart of the proposed method for the extraction of the daily new feeding trails.

The daily new feeding trails were extracted using the output of section 2.4 (Fig 8). Extraction of the daily new feeding trails was based on a differential image generated from two output images from the Model_1. One was an output image from a certain day, and the other was that from the day before. If the image of the day before was not available, the image for the latest (2 to 5 days before) day was used. The position of the past image was corrected in two block processes, (1) large blocks stage (3200 × 3200 pixels) and (2) small blocks stage (256 × 256 pixels). Then, the differential image was generated from the image for a certain day and the corrected image for the day before. The differential image was used for the training of the Model_2 based on U-Net to extract the daily new feeding trails.

#### 2.5.2 Preprocess

Because each orthophoto had a spatial error, some errors might occur when extracting the daily new feeding trails from differential images generated without preprocessing. To estimate the spatial error of the orthophotos used in this study, orthophotos for evaluation were generated separately from those for analysis. They were generated using the coordinates of the ground-based observation areas for georeferencing. Then, the route mean square error of the GCPs’ location between pixel coordinates in orthophotos and coordinates measured in-situ were calculated. The route mean square error of the orthophotos was 1.42 ± 0.29 m (n = 10). To minimize the effect of the spatial error of each orthophoto in the subtraction process, position corrections by template matching [72] were performed on the output images of the day before as preprocess of subtraction.

First, the whole input images were split into blocks of 3200 × 3200 pixels. Then, an image of a certain day was set as the template and the image of the day before was shifted within the range of maximum offset to the location which had the highest normalized cross correlation value [73] with the template. The threshold of the offsets was set to 20% of the block length. In our study, maximum 3 m of position offsets between subsequent day datasets was corrected. The maximum offset was larger than spatial error of orthophotos (1.42 ± 0.29 m). Therefore, spatial errors of orthophotos were minimized. For the detailed position corrections, the same process was performed after the images were split into images of 256 × 256 pixels. After these processes, the differential images (256 × 256 pixels) were generated.

#### 2.5.3 Learning deep neural networks based on U-Net

**Fig 9:**
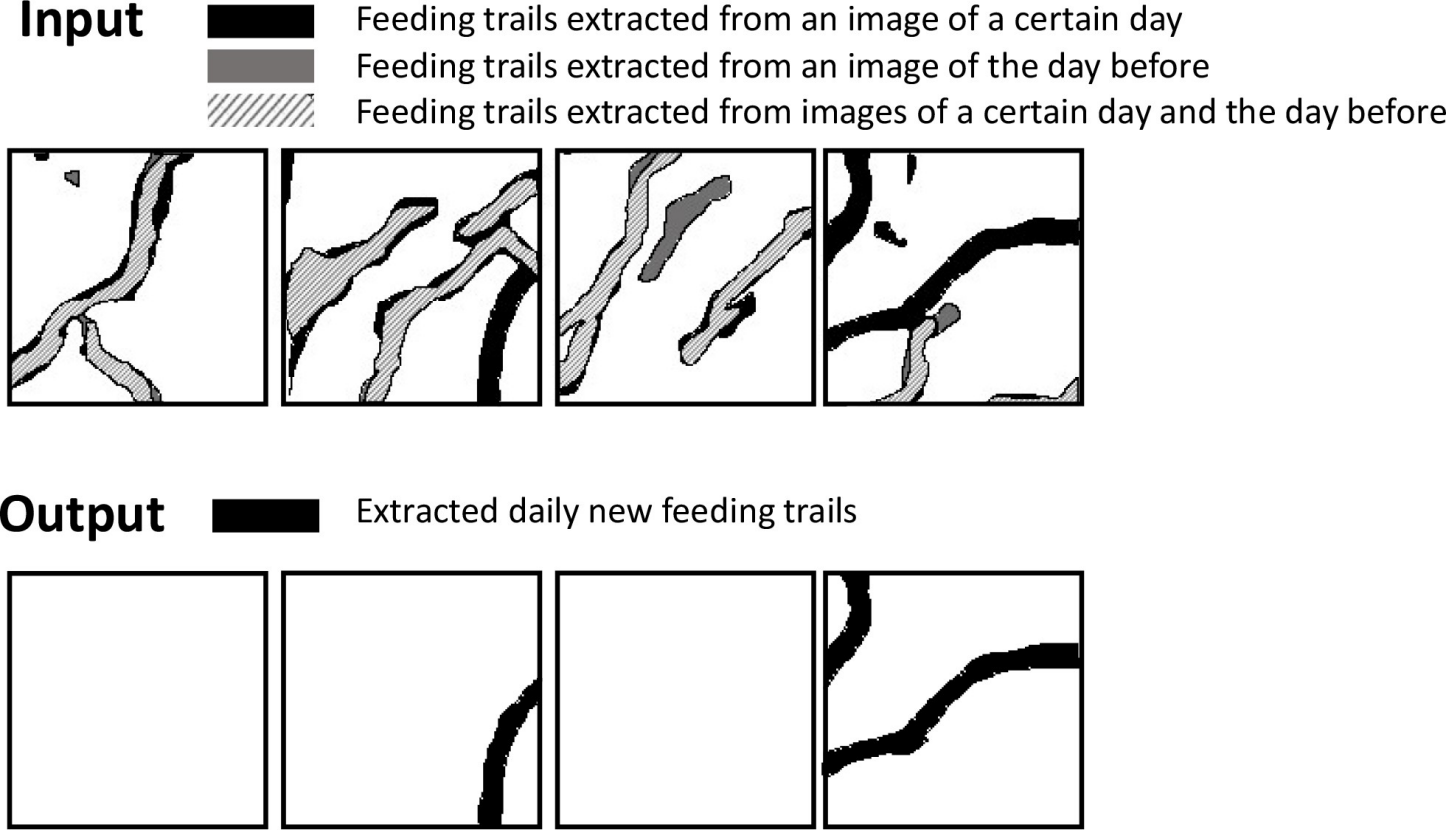
Examples of training data sets used for the extraction of the daily new feeding trails. Upper inset shows the input images. The black lines show feeding trails extracted only from an image of a certain day. The gray lines show feeding trails extracted only from an image of the day before. The striped lines show feeding trails extracted from both the image of a certain day and that of the day before. The lower inset shows the output images. The black lines in the lower inset show extracted daily new feeding trails.

The Model_2 based on the U-Net was trained to extract the daily new feeding trails from the preprocessed differential images (Fig 9). The training data set was made by manual annotation, referring to the records of the ground-truth observations. The daily new feeding trails were annotated on the orthophotos of a certain day overlayed with those of the day before. The data set consisted of 5 pairs of two days -worth of data (September 15–17, 17–18, 18–19, 20–25 and 25–26). A total of 18,000 images for the orthophotos of plot A (3600 per one pair) were used. The network architecture and the parameters were the same as those described in section 2.4.1. For data augmentation, each image was cropped to be overlapped by 75% (192 pixels) with the neighboring images. The cropped images were augmented by random rotation (-90, 0, 90, 180) and flip.

#### 2.5.4 Extraction of the daily new feeding trails

The preprocessed images were fed to the Model_2. The predicted results were integrated and binarized by the luminance value threshold. The threshold was determined to be 125. Then, the morphological opening filter was used to remove the noise. The morphological opening filter was the morphological erosion followed by the morphological dilation. In the dilation operation, the structure element was positioned at all possible locations, and its origin (the center of the shape) was set to 0 if any of its corresponding neighborhood pixels were also 0. The F1 score was also used to assess the accuracy of the Model_2.

### 2.6 Estimation of the direction of the feeding trails

To visualize detailed movement during the dugongs’ feeding, an estimation algorithm for the direction of the feeding trail was developed. The binary image of the aerial observation area, which is the output of the Model_1 was used as an input. The input image was divided into blocks of 256 × 256 pixels, and then eight directions (0°, 22.5°, 45°, 67.5°, 90°, 112.5°, 135°, and 157.5°) of the extracted area in each block were estimated. Estimations were based on the likelihood of each direction as stated below. The blocks in which the extracted area was less than 8% of their total area were excluded from the estimations.

**Fig 10:**
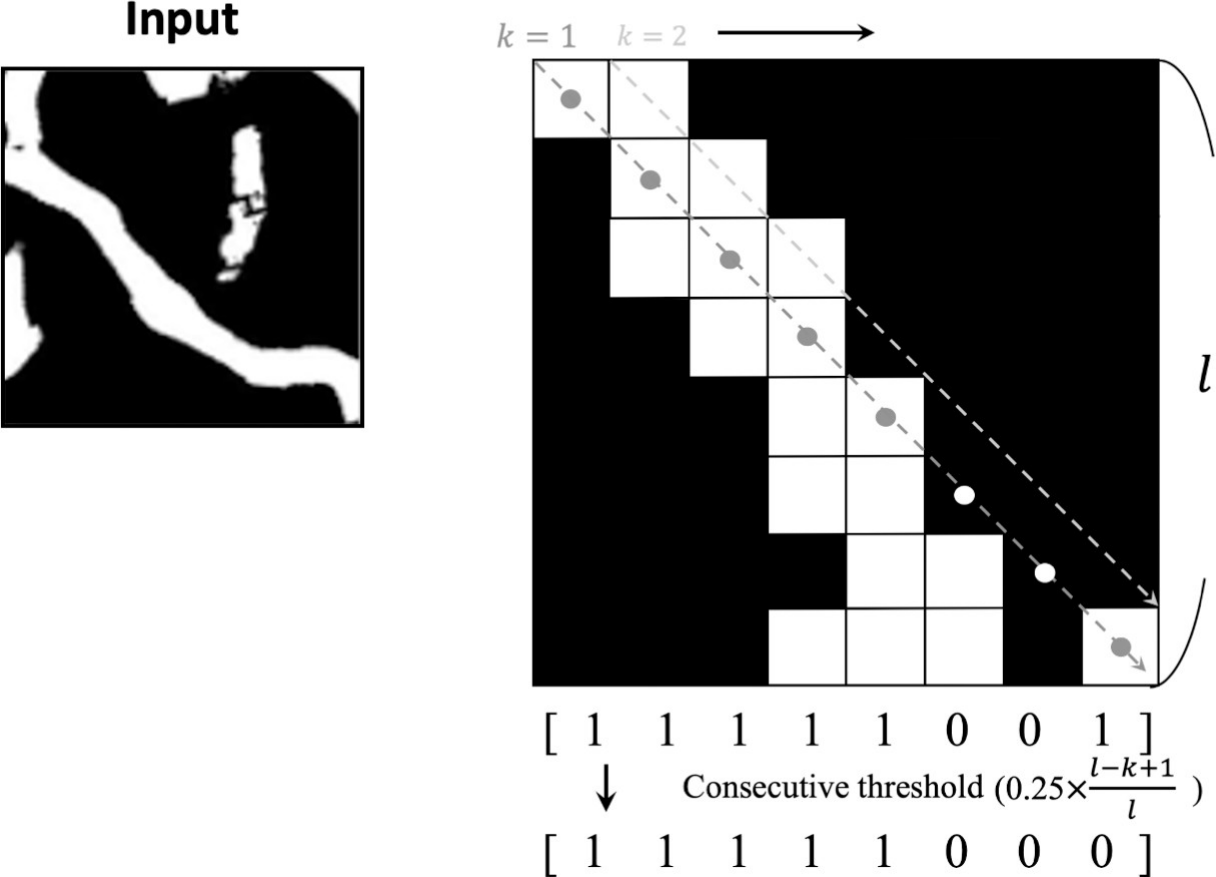
An example of the inputted matrix of 256 × 256 pix (left inset) and the explanatory figure of the estimation of the feeding trails’ direction (right inset). Right inset shows an example of calculating likelihood of 135°. The white pixels show the area extracted as the feeding trails; element 1, and the black pixels show the background; element 0. The gray and white dots show the coordinates of elements 1 in the matrix *M*_1_. The row vectors *v*_1_ and *c*_1_ are shown in the lower inset; *v*_1_ contains the sum of each column of *M*_1_ × *O*_1_ and was converted into *c*_1_, of which the non-consecutive element 1 within the threshold of 0.25(*l*−1)/*l* was replaced with 0.

An example of calculating the likelihood of 135° is shown in Fig 10. The one side of the inputted matrix *O* is shown as *l*. In *O*, the extracted area has the element 1. The first, matrix *M*_*k*_ was indexed by *k* (−*l*+1≤*k*≤*l*) that had the element 1 and the specific direction was created. *M*_*k*_ had element 1 in coordinates (*k*−1,0). For the 135° example, a diagonal matrix was created. Then, a row vector *v*_*k*_ containing the sum of each column of *M*_*k*_ × *O*_*k*_ was calculated. The non-consecutive element 1 in the *v*_*k*_ within the threshold of 0.25(*l*−*k*)/*l* was replaced with 0. This converted vector was defined as *c*_*k*_. This process aimed to exclude the effects of the noise and feeding trails in different directions. The likelihood of certain directions, defined as $\sum_{k=-l+1}^{l}\sum c_{k}$ were calculated for each direction, and then, the direction with the highest likelihood was defined as the direction of the feeding trail in an inputted matrix as *O*.

## 3 Results

### 3.1 Automated extraction of the feeding trails

**Fig 11:**
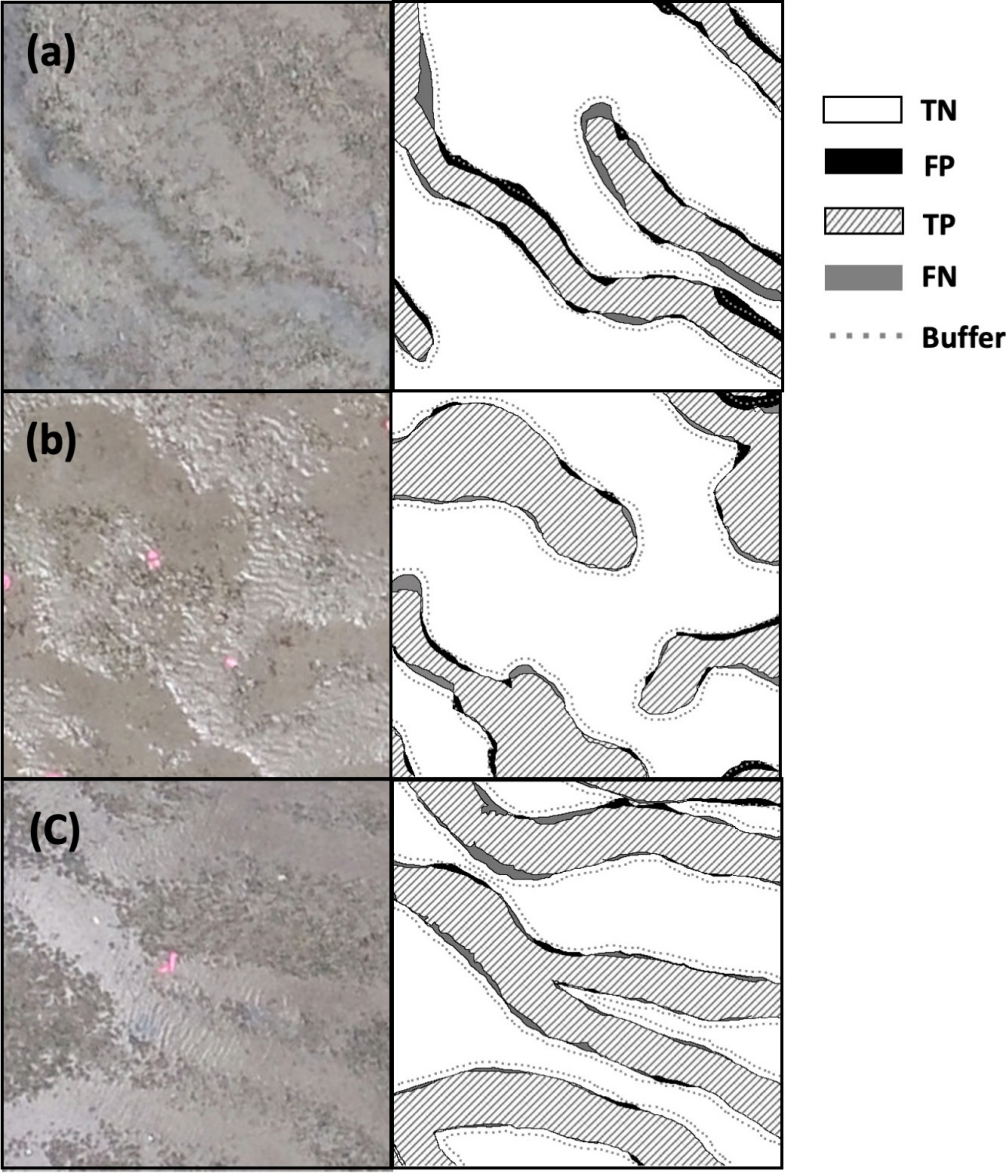
**Examples of input (left inset) and extraction (right inset, true negatives TN are in the white area, false positives FP in the black area, true positives TP in the striped area, and false negatives FN in the gray area).** The gray dotted line shows the buffer boundaries for the 7 pixels (0.47 cm/pixel) used in this study.

The F1 score of the Model_1 with the buffer of 7 pixels was 89.5% and 87.7% in the images with a ground sampling distance of 1 cm/pixel and 0.5 cm/pixel, respectively (Table 2). The extraction performance was robust for various illuminances or colors, as shown in Fig 11. The feeding trails with multiple color variations were extracted correctly (Fig 11A). They were also correctly extracted in both conditions so that the luminance of the feeding trails area was lower (Fig 11B) and higher (Fig 11C) than that of the surrounding area.

**Table 2:** Feeding trail extraction for the input images of different ground sampling distances (precision, recall, and F1 score).

Ground samping distance (cm / pixel)	Precision (%)	Recall (%)	F1 score (%)
1	96.8	83.2	89.5
0.5	94.4	81.8	87.7

**Fig 12:**
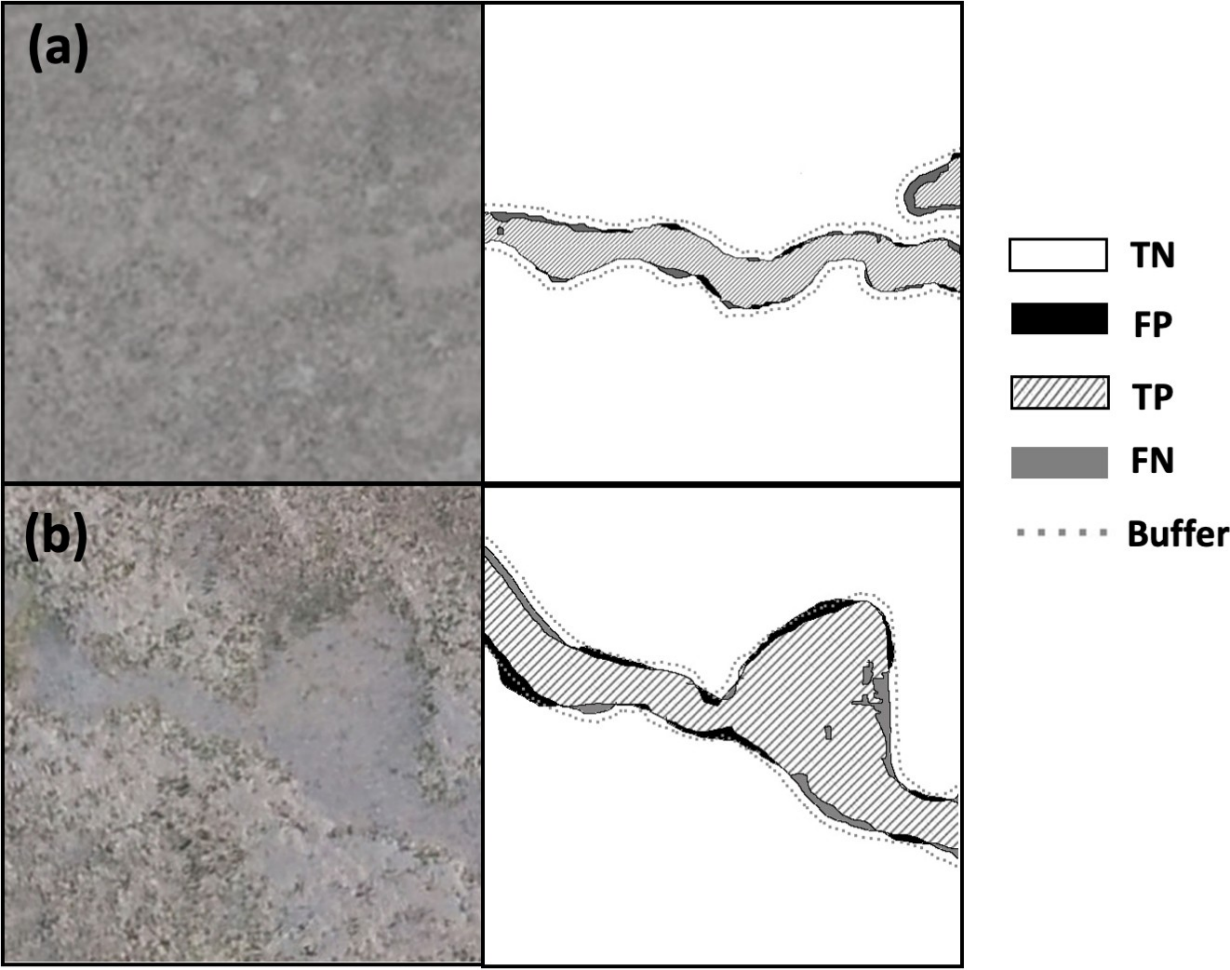
Examples of input (left inset) and extraction (right inset, true negatives TN are in the white area, false positives FP in the black area, true positives TP in the striped area, and false negatives FN in the gray area). The gray dotted line shows the buffer boundaries for the 7 pixels (0.47 cm/pixel) used in this study.

Feeding trails on the images with coarser ground sampling resolutions of 1 cm/pixel (Fig 12A) and some feeding trails with abrupt changes in width (Fig 12B) were correctly extracted.

**Fig 13:**
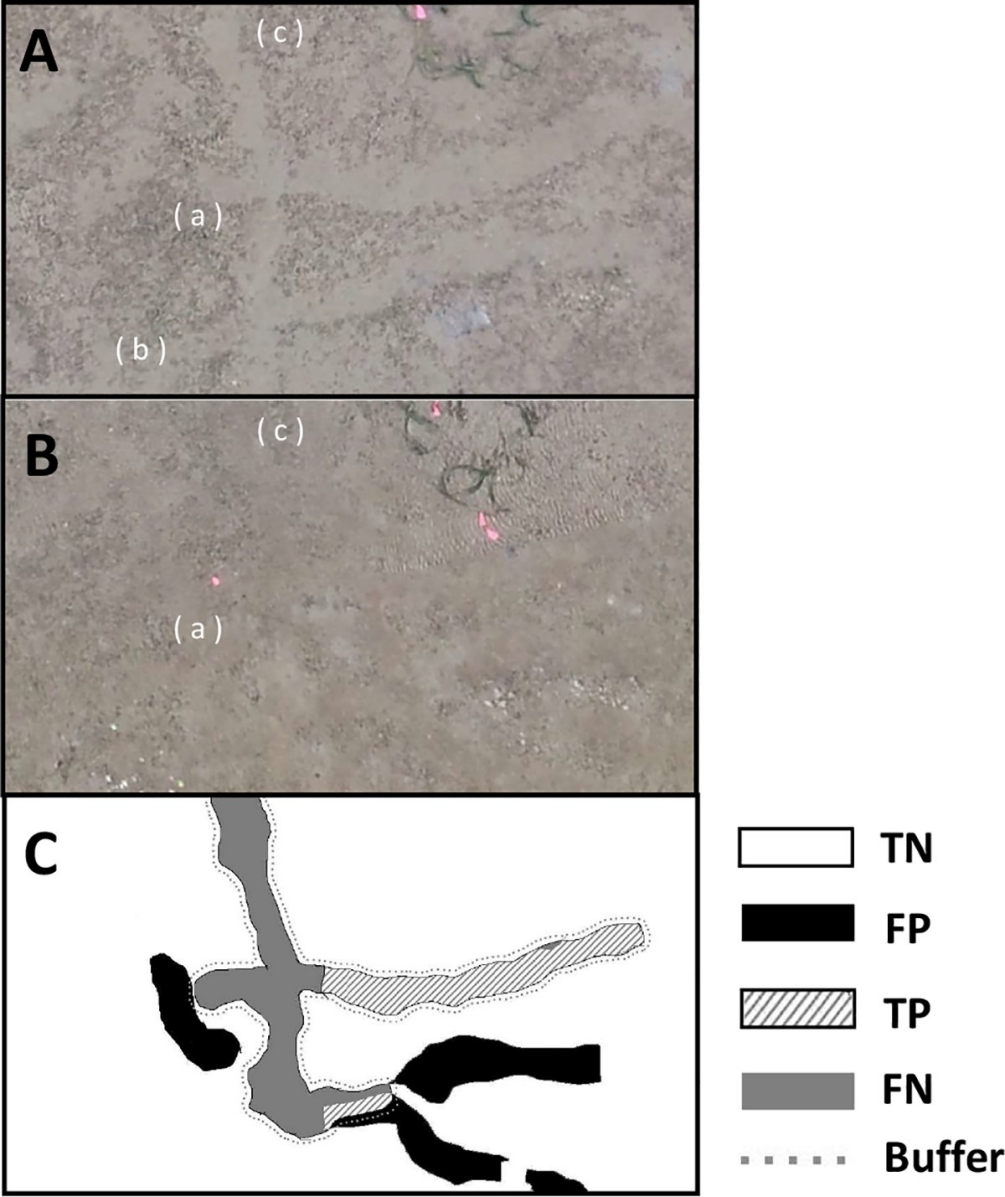
Examples of the extraction errors; (A) orthophoto of a certain day and (B) orthophoto of 10 days later, (C) extraction of (B). True negatives TN are in white, false positives FP in black, true positives TP in the striped area, and false negatives FN in the gray area. The gray dotted line shows the buffer boundaries of the 7 pixels (0.47 cm/pix) used in this study.

Errors represented by FP and FN occurred when extracting the old feeding trails. For example, 3 feeding trails were observed (Fig 13A(a)–13A(c)) on the orthophoto of a certain day. Ten days later, it was impossible to distinguish (b) from the surrounding area on the orthophoto (Fig 13B). However, part of (b) was extracted by the Model_1 (Fig 13C) and (c) was not extracted.

### 3.2 Automated extraction of the daily new feeding trails

**Fig 14:**
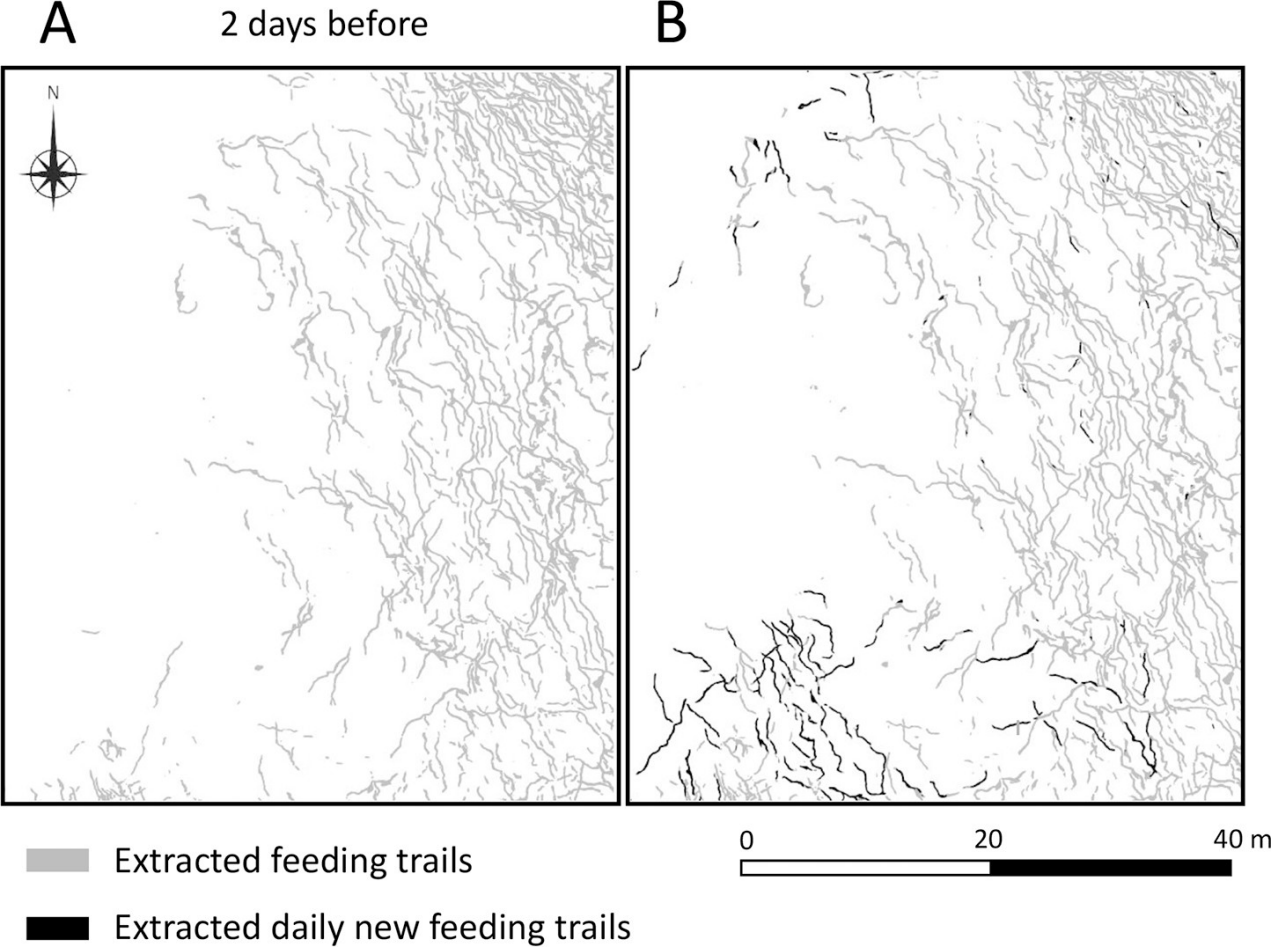
Example of the extracted daily new feeding trails; (A) feeding trails extracted from the orthophoto image of 2 days before and (B) feeding trails extracted from the orthophoto image of a certain day. The gray area shows feeding trails extracted by the Model_1. The black area (B) shows new daily feeding trails extracted by the Model_2 and is overlayed on the feeding trails extracted by the Model_1.

The F1 score of the Model_2 were 61.9%. The extracted daily new feeding trails were demonstrated in Fig 14. The errors were manually corrected. The estimated area for the average daily new feeding trails from September 12 to 27 was 187.8 m^2^ per day (n = 9).

### 3.3 Estimation of the direction of the feeding trails

**Fig 15:**
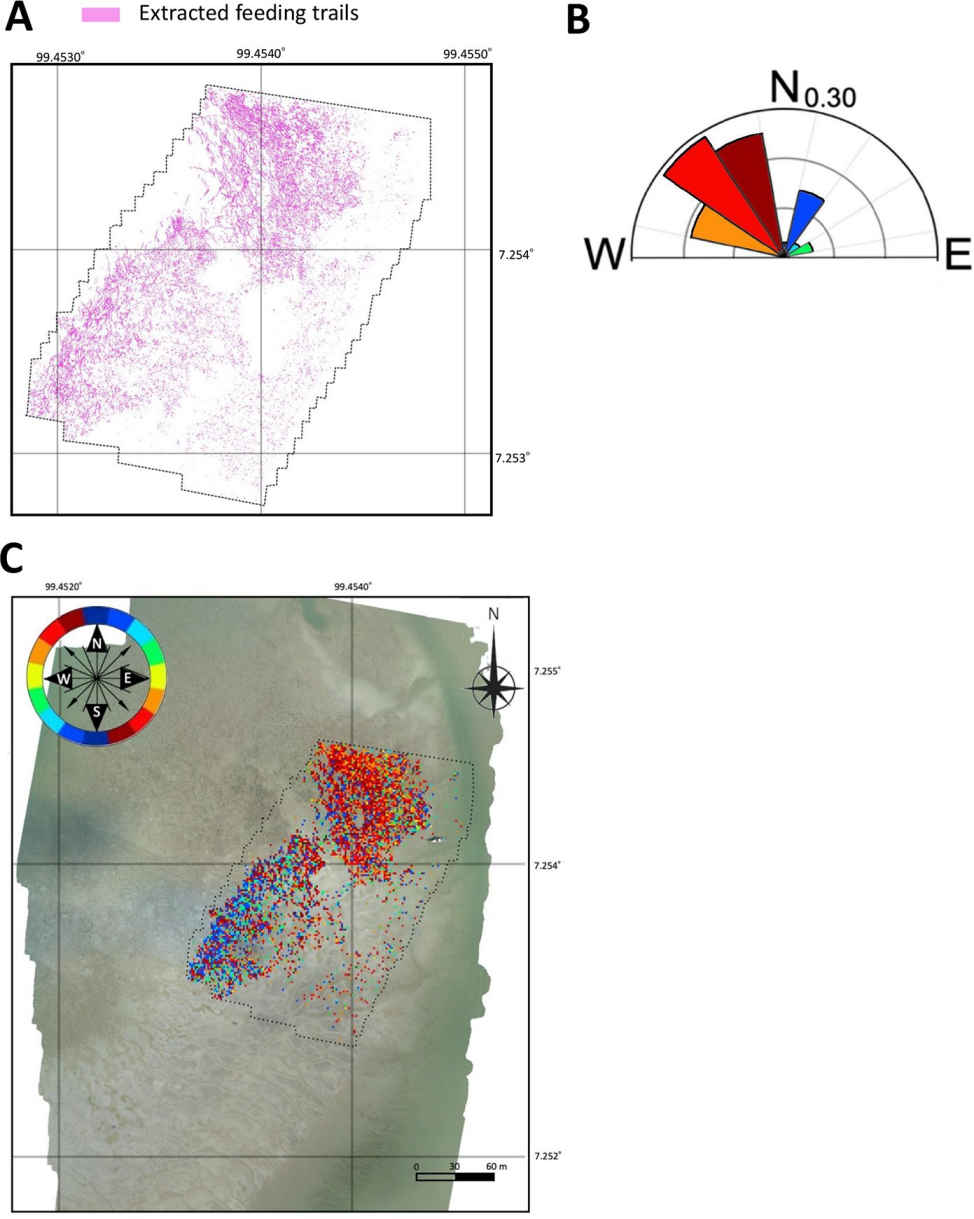
Example of the (A) input and (B) (C) output from the estimation algorithm for the feeding trails’ direction. The pink lines in (A) show feeding trails extracted by the Model_1. (B) shows the circular histogram of the estimated direction. In (C), each block of 256 × 256 pixels within the aerial observation area (dotted line) was classified into 8 colors according to the estimated direction (0°, 22.5°, 45°, 67.5°, 90°, 112.5°, 135°, and 157.5°, and the corresponding legend is in the upper left of the feeding trails).

Of the extracted feeding trails, 63.9% was estimated to have direction within a range of 112.5° and 157.5° and 17.7% was estimated to have a direction of 22.5° (Fig 15).

## 4 Discussion

The feeding trails were observed efficiently using the proposed method, that included a UAV survey and automatic feeding trail extraction with deep neural networks.

UAV surveys allowed for low-cost and large-scale data collection in comparison with ground-truth observation. It took 8 days and 7 days to record all the feeding trails in plot A and plot B, respectively, for ground-truth observations. Daily ground-truth observation was conducted by 5 to 7 observers for approximately 3 hours. In contrast, UAV surveys were completed in 2 hours and conducted by a single operator. In addition, the area of each ground-truth observation area was 0.09 ha, and that of the aerial observation was 3.6 ha.

The ground sampling distances were 0.5 and 1.0 cm/pixel, and the extraction performance was found to be similar at both resolutions (Table 2). In this study, a flight designed for 1.0 cm/pixel (at the altitude of 37.7 m) was calculated to cover a 3.81 times wider area than that of the 0.5 cm/pixel (at the altitude of 18.9 m) within the same flight time. It is indicated that data collection at a ground sampling distance of 1.0 cm/pixel allows larger-scale observations without compromising the extracting performance of the feeding trails. However, an optimal ground sampling distance for the dugong study needs to be further explored. At the proposed resolutions in this study, it may be possible to obtain the distributions of seagrass coupled with those of the feeding trails. Although seagrass monitoring based on UAV survey is increasing [41–44], few case studies on monitoring small species such as *Halophila ovalis* dominated in this study site [74]. The simultaneous monitoring of the feeding trails and seagrass will be a future task. This will promote studies on the dugongs’ feeding selectiveness.

In addition, one of the most important advantages of UAV survey is that it allows for surveys with optional frequencies. Therefore, daily, tidal and seasonal dynamics could be monitored depending on the research purpose. The long-term spatial changes in the seagrass habitats of sirenians have previously been documented. For example, the rotational grazing of dugongs, which intensively feed on seagrass bed as a seasonal feeding ground and then move on to allow the seagrass to recover, has been reported [4]. Rotational grazing within multiple intertidal seagrass beds [27] and the annual presence in the seagrass habitat of the manatee has also been reported [75]. Additionally, the proposed method can be applied to estimate seagrass consumption for a certain period of time when seagrass biomass is evaluated. Seagrass consumption is estimated considering the areas of the feeding trails made in a certain period and the seagrass biomass removed from that area [26, 28]. Such information has important implications for the spatio-temporal management of the dugongs.

The deep neural networks allowed effortless analysis with high accuracy which is enough to perform scientific studies on the dugong. The Model_1 provided useful results considering other remote sensing works on seagrass based on high-resolution UAV images. For example, Duffy et al. [42] achieved 16.12% and 9.45% of root mean squared deviation in classifying *Zostera noltii* in the two intertidal seagrass beds using unsupervised optical classification. Nahirnich et al. [44] achieved an accuracy of 91.5% in classifying submerged eelgrass. Additional spectral layers may improve the extraction performance because James et al. [43] demonstrated that classifications of *Zostera marina* with Maximum Likelihood Algorithm showed high overall accuracy for the RGB benchmark (93.64%) and gains when the spectral bands were added (red edge (RE)+ near-infrared (NIR) contributions: +4.46%). In addition, new segmentation algorithms using global context information (e.g. DeepLab v3 [76]; PSPNet (Pyramid Scene Parsing Network [77]) are emerging. In this case, the U-Net architecture was employed because it is computationally efficient and delivers good results with small amounts of reference data, and the proposed method already provided useful results. The next step will include testing these approaches which may improve our method. However, there are limitations to the improvement of accuracy. It is frequently stated that visual estimation of plant coverage is no less susceptible to observer bias [78, 79]. Similar issues might have caused errors in this study. For example, old feeding trails as shown in Fig 13C, were difficult to perceive and caused some errors.

The daily new feeding trails were also efficiently observed. However, the F1 score of the Model_2 should be improved, and there remains a need for manual correction. The Model_2 had cumulative errors from the Model_1 and generation of orthophotos. In this study, we used a handy GPS to measure the GCPs positions. The orthophoto’s error would be reduced by changing the positioning method of the GCPs. In the field of fine-scaled UAV monitoring, Barry and Coakley [80] achieved 0.41 cm of horizontal error under field conditions throughout a 2-ha site, with ground resolution of 1.17 cm/pixel. Duffy et al. [42] achieved 0.32 pixels of error with ground resolutions of 0.43 cm/pixel in the seagrass bed. Most of these studies collected the GCPs using a Differential Global Positioning System (DGPS) or Real Time Kinematic Global Positioning System (RTK-GPS). Therefore, future work should consider the GCPs’ positioning method.

The dominant directions of the feeding trails seemed to differ by locations. Three of the most frequent directions were within the range of 112.5° and 157.5° (total 63.9%) and the feeding trails in these directions were concentrated in the northern half of the aerial observation area (Fig 15C). The fourth most frequent direction was 22.5° (17.7%) and the feeding trails in this direction were concentrated in the southwestern part of the area. It is possible that the dugongs’ feeding directions were influenced by the dominant tidal currents that changed according to the geographical conditions and the time of day, although the tidal current direction and feeding timings were not measured in this study. The tidal cycle’s effects on dugong movement has been previously reported [13, 21]; however, the detailed movements during feeding remain unknown.

The proposed method based on UAV and deep neural networks will reduce the time and effort required for long-term observations in multiple intertidal seagrass beds and thus contribute to a better understanding of the dugong feeding behavior.
